# Neuroprotective Actions of Dietary Choline

**DOI:** 10.3390/nu9080815

**Published:** 2017-07-28

**Authors:** Jan Krzysztof Blusztajn, Barbara E. Slack, Tiffany J. Mellott

**Affiliations:** Department of Pathology and Laboratory Medicine, Boston University School of Medicine, 72 East Concord Street, Boston, MA 02118, USA; bslack@bu.edu (B.E.S.); tmellott@bu.edu (T.J.M.)

**Keywords:** Alzheimer’s disease, autism, brain, choline, epilepsy, DNA methylation, memory, nutrition, pregnancy

## Abstract

Choline is an essential nutrient for humans. It is a precursor of membrane phospholipids (e.g., phosphatidylcholine (PC)), the neurotransmitter acetylcholine, and via betaine, the methyl group donor *S*-adenosylmethionine. High choline intake during gestation and early postnatal development in rat and mouse models improves cognitive function in adulthood, prevents age-related memory decline, and protects the brain from the neuropathological changes associated with Alzheimer’s disease (AD), and neurological damage associated with epilepsy, fetal alcohol syndrome, and inherited conditions such as Down and Rett syndromes. These effects of choline are correlated with modifications in histone and DNA methylation in brain, and with alterations in the expression of genes that encode proteins important for learning and memory processing, suggesting a possible epigenomic mechanism of action. Dietary choline intake in the adult may also influence cognitive function via an effect on PC containing eicosapentaenoic and docosahexaenoic acids; polyunsaturated species of PC whose levels are reduced in brains from AD patients, and is associated with higher memory performance, and resistance to cognitive decline.

## 1. Choline: An Essential Nutrient for Humans

Choline is an essential nutrient; that is, together with essential amino acids, fatty acids, vitamins and minerals, it must be obtained from the diet to maintain health [1]. Because choline is present in many foods, a variety of diets can satisfy the need for this nutrient. For the purposes of estimating choline intake by people, researchers use a database available at the US Department of Agriculture web site that lists dietary compounds that contain the choline moiety (i.e., free choline, glycerophosphocholine, phosphocholine, phosphatidylcholine, sphingomyelin and betaine—a metabolite of choline) [2]. Because all of those compounds provide bioavailable choline, studies on choline intake generally refer to the total dietary choline. Adequate Intake (AI) recommendations, issued by the Food and Nutrition Board of the Institute of Medicine of the National Academy of Sciences, call for an average daily intake of 7.5 mg of choline per kg of body weight, but are higher during pregnancy and lactation [1], given the needs of the fetus and nursing baby [3,4,5]. Reported pathological effects of low choline consumption include liver dysfunction in adult men [6], muscle damage [7], and apoptotic death of lymphocytes [8]. Moreover, higher consumption of choline or betaine (an oxidized choline metabolite and a methyl group donor) in adults is associated with reduced risk of some cancers [9,10,11,12,13] (reviewed in [14]).

Choline has been shown to exert neuroprotective effects in both animal and human studies. High choline intake during the perinatal period is neuroprotective in a variety of animal models of neuronal dysfunction, including that resulting from aging [15,16,17], seizures [18,19,20,21], prenatal alcohol exposure [22,23,24,25,26,27] and the genetic disorders Rett syndrome [28,29,30,31] and Down syndrome [32,33,34,35,36,37,38]. Dietary supplementation with choline-containing compounds, alone or in combination with other methyl donors such as folate and vitamin B12, was also found to ameliorate neurological impairments in animal models of stroke [39], and ischemia [40]. Moreover, high choline consumption during pregnancy correlated with reduced risk of neural tube defects in humans [41,42]. This association was less robust in more recent studies—possibly as a result of the addition of choline to vitamin supplements routinely taken by pregnant women, and variations in total daily caloric intake and reported ranges of choline consumption, in the different studies [43,44]. Dietary intake of choline in adult human subjects has been shown to correlate with cognitive function [45], and treatment with certain choline-containing compounds showed a limited tendency to reduce cognitive impairment in human vascular dementias in small clinical trials [46].

In this review, we summarize the results of studies in animal models and humans on the effects of perinatal choline nutrition on brain development, on cognitive function in the adult, and on the progression of several neurodegenerative diseases. We also discuss emerging evidence for neuroprotective effects of dietary choline consumption during adulthood. While we focus on choline, it is important to recognize that other nutrients such as folate, vitamin B12 and vitamin B6, influence the metabolism of choline, and their availability may modify the requirements for choline.

## 2. Choline Nutrition during Development and Cognitive Function

### 2.1. Protection against Age-Related Memory Decline and Advancement of Hippocampal Development

**Figure 1:**
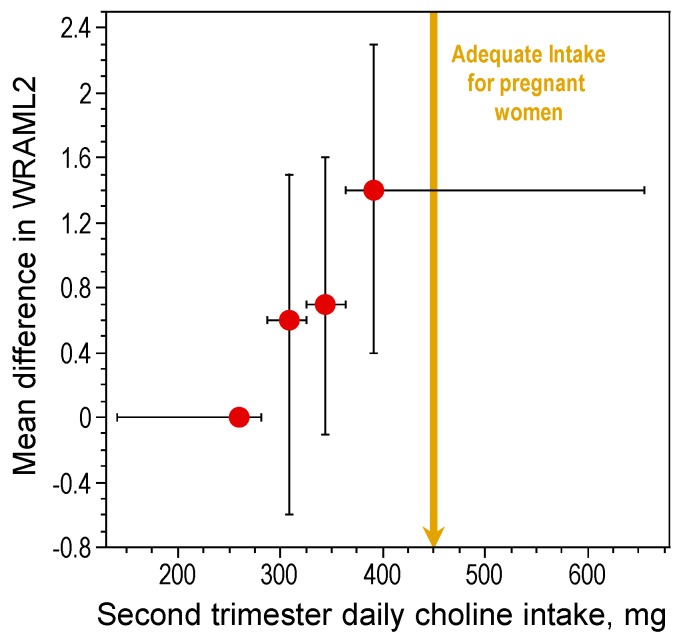
Relationship between intake of choline during the second trimester of pregnancy and offspring visual memory assessed by Wide Range Assessment of Memory and Learning, Second Edition (WRAML2). The top quartile choline intake was associated with a child WRAML2 score 1.4 points higher than the bottom quartile (P-trend = 0.003). Figure created from data in [55].

In rats, high maternal choline consumption at specific times during pregnancy (Embryonic Days 11–17) enhances cognitive ability of the offspring throughout life [15,17,47,48,49,50,51,52,53]. One important developmental benchmark, the ability to navigate using relational cues in a Morris water maze, was found to occur three days earlier in prenatally choline-supplemented rats [51]. The acquisition of this ability is thought to signal the onset of hippocampal function [54]. Thus, prenatal choline supplementation causes an approximately three-day advancement in hippocampal development. While it is not possible to conduct a similar, well controlled study in humans, data obtained by project Viva in Massachusetts show that maternal choline intake within the AI range during pregnancy was associated with better memory function in children at seven years of age as compared to children of mothers whose consumption was approximately 50% of the AI levels [55] (Figure 1). Thus, in humans, as in animal models, higher gestational consumption of choline has long-term positive effects on cognition during childhood. However, it is not yet known if these effects persist into adulthood and old age in humans. Using a rat model, Meck et al. [17] altered choline availability to rats during seven timeframes spanning Embryonic Day (E) 6 through Postnatal Day (P) 75 and examined spatial memory ability in the perinatally-treated adults. Two sensitive periods were identified, E12–17 and P16–30, during which choline supplementation facilitated spatial memory. Moreover, choline supplementation during E12–17 only, prevented the memory decline normally observed in aged (26 months old) rats [17].

### 2.2. Cellular, Transcriptomic, Proteomic and Epigenetic Correlates of the Neuroprotective Action of Choline

During fetal development, maternal choline deficiency inhibits hippocampal precursor cell proliferation and stimulates apoptosis in the hippocampus [56], whereas gestational choline supplementation stimulates hippocampal cell division [57]. In the adult, neurogenesis in the dentate gyrus of the hippocampus, a process that occurs throughout life [58,59,60], is enhanced by prenatal choline supplementation, and impaired by prenatal choline deficiency [20,61,62]. This effect on adult neurogenesis was associated with increased hippocampal concentrations of the trophic factors nerve growth factor (NGF), brain-derived neurotrophic factor (BDNF), insulin-like growth factor 2 (IGF2), and vascular endothelial growth factor (VEGF) [20,61,62,63,64,65], increased size of basal forebrain cholinergic neurons [66]—cells required for normal cognitive function [67,68]—and elevated acetylcholine synthesis and release from these neurons [17,65,69]. Prenatal choline supplementation increased the phosphorylation of mitogen-activated protein kinase (MAPK) and 3′,5′-cyclic adenosine monophosphate (cAMP) response element binding protein (CREB) in response to activation of glutamate receptors in hippocampus, [51]. The MAPK signaling cascade, and its downstream targets including CREB, are central players in the processes underlying synaptic plasticity, and learning and memory [70]. The induction in hippocampus of long term potentiation, an electrophysiological correlate of synaptic plasticity [71], was similarly sensitive to prenatal choline availability [49,72]. Gene expression analysis identified 530 hippocampal and 815 cerebral cortical mRNA species whose levels were affected by prenatal choline status [64]. The protein products of a subset of these genes, including calcium/calmodulin-dependent kinase 1 and IGF2, participate in signaling pathways involved in memory processes and thus may contribute to the observed choline-induced changes in cognitive performance (reviewed in [73]).

Advances in the field of epigenetics have provided a framework to explain how heritable changes in gene expression patterns may be propagated during development and throughout adulthood without altering the primary DNA sequence. The methylation of CpG sequences within the regulatory elements of genes changes their expression via an interaction with a complex network of proteins, including transcription factors [74]. Following DNA replication, the unmethylated daughter strand in hemimethylated DNA is symmetrically methylated by the enzyme DNA methyltransferase 1 (DNMT1) [75], thus recapitulating the parent methylation pattern in the daughter cells. The process of DNA methylation is dynamic [76] and responsive to changes in the environment, including alterations in nutrient availability. In particular, DNA methylation is modulated by the availability of nutrients that serve as methyl group donors and cofactors, such as choline, betaine, methionine, folic acid and vitamin B12, via their influence on tissue levels of *S*-adenosylmethionine (SAM) (the methyl group donor for most enzymatic methylation reactions) (see [77] for a review). Kovacheva et al. [78] evaluated DNA methylation parameters in liver and cerebral cortex on E17 in rats exposed in utero to varying amounts of choline (via the diet supplied to pregnant dams) beginning on E11. The investigators focused on the differentially methylated region 2 (DMR2) of the *Igf2* gene, which undergoes a dramatic change in methylation status during development [79]. DMR2 methylation was increased in choline-deficient embryos, as compared to the control and choline-supplemented rats, and was accompanied by a compensatory increase in the expression of the maintenance DNA methylase *Dnmt1*. Because DNA methylation is highly dynamic in adult brain and may modulate the expression of genes involved in the regulation of synaptic plasticity [80,81,82,83,84] and learning and memory [85,86,87,88,89,90,91,92,93,94,95,96], it is possible that dietary availability of methyl donors such as choline in the adult will also prove to be an important determinant of cognitive function.

### 2.3. Neuroprotective Actions of Perinatal Choline in Animal Models of Disease

#### 2.3.1. Epilepsy

Status epilepticus, a period of prolonged seizures, is a neurological condition that produces multiple degenerative and regenerative changes in the hippocampus [97]. These changes are accompanied by cognitive deficits in hippocampal-dependent tasks [97]. Several studies tested the effects of high choline intake in rat models of chemically-evoked epilepsy. In pilocarpine- [18] and kainic acid- [19,21] induced status epilepticus, prenatal choline supplementation attenuated the deficits in visual-spatial memory. In addition, these protective actions of choline were accompanied by a decrease in seizure-induced hippocampal neurodegeneration, and a reduction in compensatory proliferation of cells within the dentate gyrus [98]. Moreover, choline supplementation prevented hippocampal loss of GAD65 mRNA [98], which encodes a form of the γ-aminobutyric acid (GABA)-synthesizing enzyme, glutamic acid decarboxylase, that is localized to the nerve terminals. As in the other rodent models [20,29,30,61,65,98,99], prior to seizures choline supplementation also increased the levels of several trophic factors in the hippocampus: specifically, NGF, BDNF and IGF1, indicating that this nutritional treatment may establish a neuroprotective hippocampal milieu that attenuates the neuropathological response to and/or helps facilitate recovery from seizures to protect cognitive function.

#### 2.3.2. Down Syndrome

Down syndrome, a common form of mental retardation, affects approximately 1 in 700 births in the United States [100]. The disorder is caused by trisomy of the whole or a part of chromosome 21, and the resulting increase in expression of the genes encoded on the extra chromosome [101]. Strupp and colleagues [32,33,34,35,36,37,38] used a mouse model of Down syndrome (Ts65Dn mice) to test the hypothesis that choline supplementation from conception to weaning could prevent some of the neurological and cognitive deficits observed in these mice. The genome of Ts65Dn mice was engineered to carry a third copy of the distal region of mouse chromosome 16, which contains approximately 94 genes orthologous to the Down syndrome critical region of the human chromosome 21 [102]. The adult offspring of choline-supplemented Ts65Dn dams performed significantly better than control Ts65Dn mice in several visual attention tasks [32,35,36]. In some of these tasks, the choline-supplemented Ts65Dn mice did not differ from the wild type controls [32]. The improvement in spatial cognition correlated with normalization of hippocampal neurogenesis in choline-supplemented Ts65Dn mice [35]. It is interesting to note that a number of genes on chromosome 21 are involved in epigenetic mechanisms, including those encoding the DNA methyltransferase-like protein DNMT3L [101], which is required for the establishment of maternal genomic imprints [103], and also stimulates *de novo* methylation by DNMT3A [104]. In frontal cortex neurons purified from Down syndrome patients and controls, 272 genes showed differential methylation of CpG islands; the majority of which were hypermethylated in Down syndrome relative to controls [105,106]. An earlier report demonstrated that global DNA methylation, measured at E17, was reduced in frontal cortex of perinatally choline-supplemented rats relative to control and choline deficient embryos, and this was reflected in a significant reduction in mRNA levels of the *Dnmt3l* and *Dnmt3a* [78] These observations raise the possibility that choline supplementation in Down syndrome models may improve cognitive function in part by opposing the hypermethylation that otherwise occurs in neurons of these animals.

#### 2.3.3. Rett Syndrome

Rett syndrome is a genetic neurological disorder of childhood that represents a common (approximately 1 in 10,000 births) form of mental retardation and almost exclusively affects girls [31]. It is usually caused by a mutation in the X chromosome-linked methyl-CpG-binding protein 2 (*Mecp2*) gene encoding a protein with possible roles in gene transcription, chromatin organization, alternative splicing, and miRNA processing [107]. Several mouse models with inactivating mutations of *Mecp2* have been developed. These mice resemble the human disease in that they are apparently normal at birth, but develop severe neurological symptoms within weeks. In a series of studies, Berger-Sweeney and colleagues tested the effects of perinatal choline supplementation in two mouse models of Rett syndrome [28,29,30,108,109]. Rett syndrome model mice were supplemented with choline or saccharine (controls) via the mothers’ milk from birth to weaning. Choline supplementation improved motor coordination and locomotor activity of *Mecp2*-null males, and grip strength in females, but did not alter cognitive deficits in either sex [28]. These improvements in locomotor function were accompanied by increases in total brain volume in females, and in cerebellar volume in males [108]. Postnatal choline supplementation increased striatal NGF expression in both wild type and *Mecp2* null mice [29] and increased the brain levels of *N*-acetyl aspartate, a marker of neuronal integrity, in nuclear magnetic resonance spectroscopy assays [109]. These results are consistent with an effect of choline on enhanced neuronal proliferation and survival in mutant mice. In mice with a different *Mecp2* mutation, early postnatal choline treatment prevented deficits in locomotor activity [30], and restored the activity of the acetylcholine-synthesizing enzyme, choline acetyltransferase (CHAT), in the striatum. In both mouse models, choline supplementation increased mRNA expression of NGF and BDNF in the cerebral cortex and hippocampus [30,31]. Taken together, these data suggest that nutritional supplementation with choline may improve neuronal function in Rett syndrome patients and thus constitutes a potential therapy for this disease.

#### 2.3.4. Schizophrenia

To evaluate the possibility that perinatal choline treatment could be useful in preventing certain psychiatric disorders, Stevens et al. [110] studied the effects of choline supplementation in the DBA/2 mouse strain, which is extensively used as a model of schizophrenia [111]—a disease with 60–80% heritability [112,113]. These mice resemble human schizophrenia patients in that they show reduced levels of hippocampal α7 nicotinic receptors, and deficits in sensory inhibition in response to repeated auditory stimuli [114]. DBA/2 dams were placed on control or choline-supplemented diets from mating until weaning. The offspring of dams maintained on the control diet displayed the characteristic abnormality in sensory processing that is also present in patients with schizophrenia, whereas prenatally choline-supplemented mice exhibited a normal sensory processing phenotype [110], suggesting that this nutritional treatment may reduce the risk of schizophrenia. This effect of choline was not seen in DBA/2 mice with one or zero copies of the α7 nicotinic receptor [114], suggesting that perinatal choline exerts its effect on sensory processing through an action on this receptor.

#### 2.3.5. Alzheimer’s Disease

Mellott et al. [62] used a mouse model of AD to examine the effects of choline supplementation from conception to weaning on AD pathology. This APPswe/PS1dE9 (APP.PS1; MGI ID: 3524957) AD model mouse strain was engineered to express murine *App* with the human Aβ amino acid sequence harboring mutations that cause a familial form of AD (the Swedish mutation APPK595N/M596L; APPswe) together with a mutated form of *PSEN1* (PS1 with exon 9 deleted; PS1dE9) [115]. Though no model of AD fully recapitulates the human disease [116], APP.PS1 mice are characterized by: (1) high production of amyloid Aβ peptides in brain and accumulation of amyloid plaques by 4–6 months of age [117]; (2) cognitive impairments [118,119,120,121,122]; (3) cholinergic defects [123,124,125,126,127,128]; and (4) evidence of abnormal methylation of several genes [129]. Perinatal choline supplementation significantly reduced the average number of Aβ42 plaques in both 9- and 12-month old APP.PS1 female and male mice. While the number of Aβ42 plaques increased with age in the control APP.PS1 mice, the plaque number was more stable in choline-supplemented mice, suggesting that Aβ42 synthesis, clearance, and/or aggregation may be altered in these mice to prevent additional plaque formation. A decline in cholinergic function and diminished expression of the cholinergic marker, CHAT, is apparent in aged humans and animals [130,131,132], in patients with Alzheimer’s disease (AD) [133,134,135,136], and in animal models of AD [118,123,124,125,126,127,128,135,137]. Thus, it has been postulated that abnormal cholinergic neurotransmission, due to dysfunction and/or degeneration of the septo-hippocampal cholinergic system, contributes to the memory deficits seen in advanced age and in AD [131,135,136]. At 9- and 12-months, CHAT protein levels were significantly decreased in the APP.PS1 female and male mice from the control group. Perinatal choline supplementation prevented this decrease, suggesting that choline supplementation may rescue cholinergic function in AD mice. APP.PS1 mice were also characterized by hippocampal gliosis [128,137,138,139]. This gliosis was nearly eliminated by perinatal choline supplementation. Given that activation of glial cells in AD and in AD mouse models may be initiated by Aβ peptides [140], it is possible that reduced gliosis in perinatally choline-supplemented APP.PS1 mice is secondary to the amelioration of the amyloidosis seen in these animals although it is also possible that high choline intake during development may have long-term anti-inflammatory actions in brain. While this study used an AD model caused by genes mutated in the familial human disease, the vast majority of AD cases are sporadic, with no known causes, and even though AD prevalence is alarming, affecting over 30% of individuals over 85 years of age [141], the disease does not appear to be an inevitable result of aging. Some of the factors that prevent or forestall AD may be genetic; e.g., non-carriers of the *APOE* ε4 allele [142,143,144,145] or individuals who inherit the rare *APP A673T* allele [146] may be somewhat protected. The study by Mellott et al. [62] suggests that vulnerability to AD may be modified by early-life nutrition.

#### 2.3.6. Autism Spectrum Disorder

Autism spectrum disorder (ASD) is a heterogeneous group of developmental conditions characterized by deficient social interactions, deficits in verbal and nonverbal communication, stereotyped, repetitive patterns of behaviors and interests, and cognitive inflexibility in childhood, frequently continuing into adolescence and adulthood [147,148,149,150,151]. ASD has high heritability and genetic studies identified multiple loci that may increase the risk of ASD [152]. However, ASD is not a simple genetic disorder and environmental factors such as maternal nutrition during the periconceptual period, and throughout pregnancy and nursing, may contribute to its etiology [153,154]. While autism may be uniquely human, animal models have been developed to mimic some of the components of the disorder. The inbred BTBR T+Itpr3tf/J mice display autism-like behavioral phenotypes, with deficits in reciprocal social interactions [155,156], impaired communication, and repetitive behaviors, as compared with the high sociability and low self-grooming of the reference strain C57BL/6J (B6) [157,158,159,160]. Langley et al. [161] demonstrated that maternal dietary choline supplementation in BTBR mice during pregnancy and lactation alleviated anxiety-like behaviors and improved deficits in social behavior in adolescent and adult progeny. Interestingly, the behavioral phenotype-related genetic quantitative trait loci in BTBR mice contain genes associated with cholinergic neurotransmission (*Chrna3*, *Chrna5*, and *Chrnb4*), choline (*Pcyt1b*, *Chpt1*, *Pld1*, and *Ppap2c*) and folate metabolism (*Mthfd1*, *Mthfs*, and *Slc19a1*), as well as DNA methylation (*Dnmt3l* and *Mecp2*). Note that the expression of *Dnmt3l* was modulated by choline availability in another study [78], and that mutations in MECP2 cause Rett syndrome (see above). Overall, the results suggested that high choline intake during early development can prevent or reduce deficits in social behavior and anxiety in an autistic mouse model.

### 2.4. Neuroprotective Effects of Choline Intake in Adults: Implications for Cognitive Aging and Alzheimer’s Disease

Despite choline’s recently acquired status as an essential nutrient [1], and its presence in many foods [2], at least 75% of American adults consume less than the recommended Adequate Intake levels (see above) [9,45,73,162,163]). A recent National Health and Nutrition Examination Survey (NHANES) found that only 4% of men and 2% of women over the age of 71 years meet the AI value [164]. This was also found to be the case in a report from the Framingham Heart Study (FHS) Offspring Cohort (FHS Generation 2), which measured cognitive performance and dietary choline intake in 1391 dementia-free subjects (age 61 ± 9 SD). Approximately 75% of the subjects in this study consumed less than the recommended AI levels of choline [45], as estimated using a Food Frequency Questionnaire in which participants reported on which foods they had eaten for the prior 12-month period.

**Figure 2:**
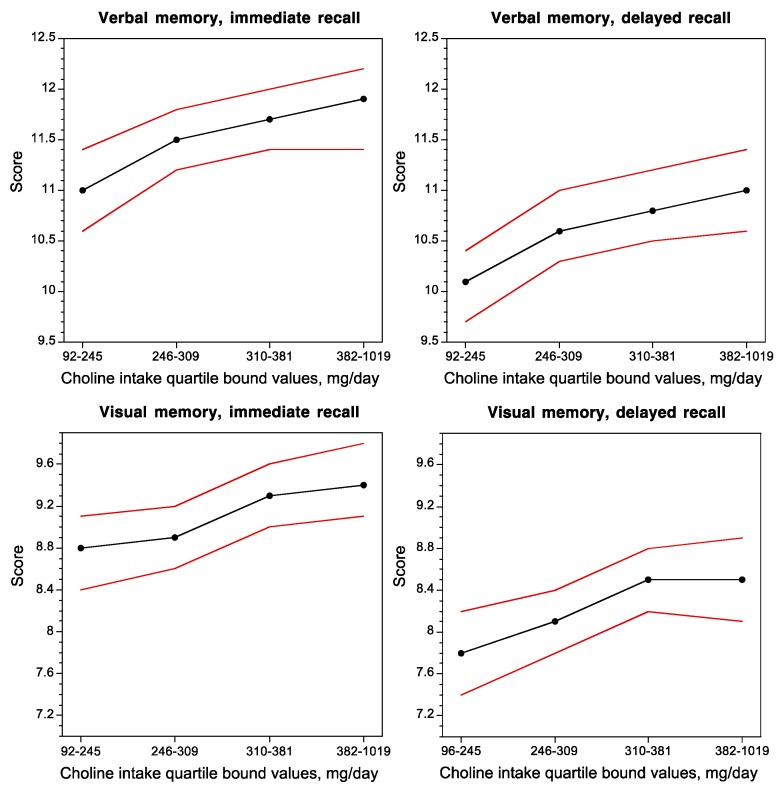
Dose–response relationship between the average daily choline intake and verbal and visual memory performance in non-demented adults. Red lines indicate 95% confidence interval. Figure created from data in [45].

Concurrent choline intake was positively correlated with the performance of subjects on verbal and visual memory tasks (Figure 2) [45], and inversely correlated with white-matter hyperintensity volume, a brain magnetic resonance imaging measurement that is associated with impaired cognitive function and Alzheimer’s disease [45].

**Figure 3:**
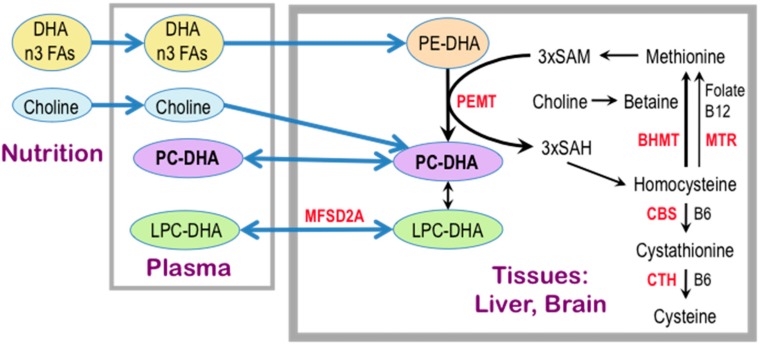
PC-DHA levels depend on nutrition, liver and brain synthesis by PEMT and transport of LPC-DHA across the blood-brain barrier catalyzed by MFSD2A. Plasma PC levels and PC FA molecular species (e.g., PC-DHA) depend on hepatic PC metabolism, the dietary supply of choline and *n-*3 FA, including DHA, and PC and LPC flux between plasma and tissues (e.g., brain). Hepatic PEMT activity is high and the liver produces large amounts of PC-DHA by methylating PE for the needs of the entire organism. This PC-DHA is secreted by the liver into the circulation as a component of lipoproteins. Brain PEMT activity is relatively low and neuronal and glial PEMT produces PC-DHA to sustain local membranes. PC synthesis by PEMT requires three molecules of SAM that is synthesized from the amino acid, methionine. In addition to PC, PEMT generates SAH that is hydrolyzed to homocysteine. The latter can be converted back to methionine by two pathways: one catalyzed by betaine:homocysteine *S*-methyltransferase (BHMT) and another catalyzed by vitamin B12-requiring 5-methyltetrahydrofolate-homocysteine *S*-methyltransferase (MTR), in which methyltetrahydrofolate is used as a methyl donor. Betaine is the product of enzymatic oxidation of choline. Thus, dietary choline is used to produce PC directly and to convert homocysteine to methionine and subsequently to SAM. In this fashion the methyl groups derived from dietary choline can be utilized by PEMT to generate PC-DHA. Recent evidence indicates that the main source of brain DHA is LPC-DHA—the circulating deacylated metabolite of PC-DHA. The transport of LPC-DHA from plasma to the brain is catalyzed by MFSD2A. The blue arrows indicate flux and the black arrows represent enzymatic pathways. Protein names are in red. Dietary DHA is primarily stored in PE in tissues and only a small fraction is incorporated directly into PC. Homocysteine can also be converted to cysteine via cystathionine in a transsulfuration pathway that includes two vitamin B6-requiring enzymes, CBS and CTH. Abbreviations: BHMT, betaine:homocysteine *S*-methyltransferase; CBS, cystathionine-beta-synthase; CTH, cystathionine gamma-lyase; DHA, docosahexaenoic acid; FA, fatty acid; LPC, lysophosphatidylcholine; MFSD2A, major facilitator superfamily domain containing 2a; MTR, 5-methyltetrahydrofolate-homocysteine *S*-methyltransferase; PC, phosphatidylcholine; PE, phosphatidylethanolamine; PEMT, phosphatidylethanolamine *N*-methyltransferase; SAH, *S*-adenosylhomocysteine; SAM, *S*-adenosylmethionine.

One possible explanation for the effect of concurrent choline intake on cognition in adults lies in its function as a precursor of the phospholipid phosphatidylcholine (PC), a major constituent of all biological membranes, including those in neurons and glial cells. Evidence that phospholipid metabolism is abnormal in AD originated with *postmortem* brain sample studies dating to the 1980s and 1990s [165,166,167,168] which showed reduced levels of PC and phosphatidylethanolamine (PE) and increased levels of their metabolites, glycerophosphocholine and glycerophosphoethanolamine, respectively, in the cerebral cortex of AD patients as compared to age-matched controls and to patients with Down syndrome, Parkinson’s disease and Huntington’s disease [167,168]. These data, and additional evidence that brain regions generally free of plaques and tangles, such as the caudate and the cerebellum, are similarly affected [167], led to the hypothesis that the phospholipid defect is specific to AD and widespread within the brain. A large body of evidence, reported since that time, is consistent with this concept (e.g., [169,170,171,172,173,174,175,176]). Moreover, there is a dramatic and specific reduction in molecular species of PC containing docosahexaenoic acid [DHA, 22:6*n-*3] (PC-DHA) levels in the temporal cortex gray matter of AD patients as compared to controls [177]. The advent of metabolomic techniques that permit untargeted large-scale analyses of hundreds of compounds combined with the intense need to discover plasma biomarkers for AD resulted in the surprising finding that lipid abnormalities in AD are not confined to the brain but are also evident in the peripheral blood plasma [176,178,179,180,181,182,183,184,185], suggesting that a blood lipidomic biomarker can be developed for prodromal AD [176,178,179,180,181,182,183,186,187], and importantly, that a lipid defect might be a driver of this disease. In particular, the plasma concentrations of PC species with 5 and 6 double bonds are consistently reported as reduced in prodromal and/or frank AD [178,181,182,183,186,188]. Data obtained by studies of erythrocyte membrane lipids further support these notions. Because erythrocytes live for an average of 120 days, their lipid profiles reflect whole body lipid metabolism over several months [189,190] as opposed to the snapshot view obtained from a plasma assay. In a study by Selley [191], erythrocytes from AD patients had reductions in concentrations of PC of 23% and of PC-DHA of 60% as compared to controls. Moreover, lower levels of erythrocyte phospholipid *n-*3 fatty acid (eicosapentaenoic [EPA, 20:5*n-*3] and DHA) content in a cohort of elderly subjects were associated with faster cognitive decline than in those with high *n-*3 FA erythrocyte content [192]. Similarly, in an elderly cohort, subjects within the lowest quartile of erythrocyte DHA concentrations had poorer memory test performance than individuals in the higher quartiles [193]. A positive correlation was observed between DHA intake and plasma PC-DHA concentrations in an FHS Generation 1 study, and was associated with a 50% reduction of dementia risk in this cohort [178]. Interestingly, in an intervention study of young women consuming 200 mg/day of DHA, increasing choline intake from 480 mg/day to 930 mg/day for six weeks increased PC-DHA concentrations in plasma and erythrocytes by 50% and 20%, respectively [194]. Conversely, plasma PC-DHA concentrations dropped in subjects placed on a choline-deficient diet [195]. Together these studies show that circulating PC-DHA levels are subject to regulation by the dietary supply of DHA and choline. Moreover, EPA and DHA are transported across the blood-brain-barrier from plasma to brain in the form of the lysophosphatidylcholines (LPC), LPC-EPA, and LPC-DHA, in a process that is catalyzed by a specific transporter, major facilitator superfamily domain containing 2a (MFSD2A) [196,197,198,199] (Figure 3). Thus, plasma levels of these LPC species may govern the rate of the supply of these essential fatty acids to brain, and there are indications of reduced plasma levels of LPC associated with AD [183,186]. Intervention studies using polyunsaturated fatty acids or DHA to treat dementia have been reviewed recently [200], and efficacy has been observed in some of those studies. Not surprisingly, early intervention works better, and the response to treatment may be attenuated in subjects with the *APOE4* genotype [201,202]—the major genetic risk factor for AD [203]. Taken together, the evidence suggests that an adequate dietary supply of choline and DHA is necessary to maintain plasma and brain PC-DHA levels, and may possibly help to slow the onset of cognitive decline with aging, and potentially to delay or ameliorate the pathogenetic process in AD.

## 3. Choline Availability, Phosphatidylethanolamine *N*-methyltransferase, and Cognitive Ability

Most of the polyunsaturated PCs in mammals, including PC-DHA, are synthesized by phosphatidylethanolamine *N*-methyltransferase (PEMT) [195,204,205,206,207], which methylates phosphatidylethanolamine (PE) to generate PC using SAM as the methyl donor (Figure 3). The main site of the PEMT-catalyzed synthesis of PC-DHA is liver [195,204,205,206], which secretes it into the circulation as a component of very low density lipoproteins. PEMT activity in brain is relatively low [208], and the polyunsaturated species of PC (including PC-EPA and PC-DHA) [207] that are synthesized there are adequate for local needs only. PEMT activity in brain is higher during the perinatal period and declines in adulthood [209]. The PEMT reaction consumes three molecules of SAM for every PC molecule produced, and generates three molecules of *S*-adenosylhomocysteine (SAH). SAH is an inhibitor of PEMT (reviewed in [210]) and so, for PEMT to be active, SAH must be rapidly hydrolyzed to homocysteine and then remethylated to generate methionine and subsequently, SAM. Alternatively, homocysteine may be catabolized to cysteine via pyridoxal phosphate (vitamin B6)-dependent transsulfuration [211]. Some of the SAH-derived hepatic homocysteine enters the circulation. This results in a quantitatively significant link between hepatic PEMT activity and plasma levels of homocysteine [212]. High blood levels of homocysteine are associated with high plasma levels of SAH [213,214], whereas low plasma SAM/SAH ratio strongly correlates with low levels of plasma PC-DHA [195], and an increased risk of AD [215]. In 2002, a prospective evaluation of dementia-free FHS Generation 1 subjects showed that elevations in plasma homocysteine concentrations over eight years prior to the onset of clinical dementia predicted the subsequent development of AD [215]. A meta-analysis confirmed these results [216]. In a study of 29 AD patients and 26 controls, the high plasma SAH and homocysteine concentrations that characterized the AD patients, were associated with reduced levels of erythrocyte PC-DHA [191]. There are two enzymatic pathways that cause the remethylation of homocysteine to methionine: one is catalyzed by the vitamin B12-requiring 5-methyltetrahydrofolate-homocysteine *S*-methyltransferase (MTR), in which methyltetrahydrofolate is used as a methyl donor, and the other is catalyzed by betaine:homocysteine *S*-methyltransferase (BHMT) (reviewed in [73]). Betaine can be derived from the diet [2] and it is also produced in the liver by choline dehydrogenase [217]. Thus, in this way, choline is not the direct precursor of the PC headgroup, but rather, once oxidized to form betaine, donates a methyl group to homocysteine to form methionine, which is then converted to SAM (Figure 3). This SAM can be used by PEMT to generate the DHA molecular species of PC. In sum, dietary choline and its metabolite betaine, as well as folate and vitamins B6 and B12, via regulation of homocysteine and methionine levels, play central roles in the maintenance of adequate levels of PC-DHA in liver, plasma, and brain, and this in turn may moderate the risk of AD in human populations.

The importance of PEMT in cognitive function is further underscored by evidence that polymorphisms in *PEMT* affecting activity may be related to AD risk. The SNP rs7946 G523A causing the amino acid substitution V175M is associated with increased risk of AD in a study of a Chinese cohort [218]. The association was particularly significant in women and individuals who are not carriers of the *APOE4* gene. In vitro, the PEMT^175M^ enzyme, encoded by the A allele associated with AD, is 35% less active than the PEMT^175V^ isoform [219], suggesting that carriers of the A allele may have a defect in PC-DHA production. Given that AD is more common in women than in men (based on FHS data, it was estimated that after the age of 65, one in five women and one in ten men are destined to develop AD [220]), the female-specific association between the rs7946 *PEMT* SNP and AD is particularly intriguing because, depending on another *PEMT* SNP (rs12325817 present in an intron), *PEMT* expression can be modulated by estrogen [195,219,221,222,223,224]. In individuals with the rs12325817 G allele, hepatic *PEMT* expression is induced by estrogen whereas in persons with the C allele this response is absent [223]. It has been proposed that during pregnancy and lactation the estrogen-induced PEMT generates PC and choline necessary for the normal development of the fetus and baby [222]. Indeed, young women on choline-deficient diets are resistant to liver and muscle dysfunction because of the high activity of their estrogen-regulated PEMT, which produces choline *de novo* [222]. In contrast, these symptoms are typically seen in men and postmenopausal women on such diets [6,225]. These observations reinforce earlier studies in animals that showed that levels of PEMT in the brain are regulated by the dietary supply of choline in a sexually dimorphic fashion, i.e., choline deficiency increases PEMT activity in brains of female, but not male, rats [226]. Thus, the production of PC-DHA can be modulated by: (1) diet; (2) intrinsic PEMT activity determined by its amino acid sequence encoded by specific *PEMT* alleles; and (3) sexually dimorphic regulation of the amount of PEMT enzyme expressed in the liver (and possibly in brain [226]) due to *PEMT* allele-specific responsiveness to estrogen. These data point to strong associations between dietary intake of DHA and choline, brain PEMT activity, sex, and phenotypes associated with cognitive impairment and AD.

Although the studies summarized in the preceding paragraphs underscore the potential benefits of adequate dietary choline intake, less is known about the potential harms of excess choline supplementation [227]. The addition of methylation pathway components such as folate, choline, and B vitamins to certain food products as well as dietary supplements has coincided with an increased incidence of diseases related to altered DNA methylation including cancer, autism spectrum disorders, and some neurological disorders (reviewed in [228]). A prospective study of male health professionals reported an increased risk of lethal prostate cancer in subjects in the highest quintile of choline consumption, even after adjusting for the presence of other nutrients that could increase cancer risk [229]. In contrast, several studies reported that high choline intake was associated with reduced incidence of breast [9], colorectal [230] and liver [13] cancer. It is clear that more studies in humans will be necessary to refine our understanding of what constitutes optimal choline intake at various stages of life, and how this may be affected by polymorphisms in the genes responsible for choline metabolism.

## 4. Conclusions

High choline intake during gestation and the early postnatal period has been shown to enhance cognitive performance in childhood, adulthood and into old age in multiple animal models and in some human studies. Moreover, choline is neuroprotective in a variety of experimental models of neuronal damage. Choline intake in adulthood may also be critical for normal cognitive function in people. The maternal choline supply during pregnancy modifies fetal DNA [78,231] and histone methylation [232], implicating an epigenomic mechanism in these long-term effects. While these epigenomic mechanisms may also operate in the adult, the effects of dietary choline both during development and in the adult brain may also be mediated, at least in part, by an influence on the peripheral and central metabolism of polyunsaturated species of PC. Taken together, the available evidence strongly supports the notion that adequate choline intake during pregnancy, and throughout life, is an important determinant of brain development, cognitive performance in the adult, and resistance to cognitive decline associated with aging and neurodegenerative disease.
